# Supplementary material for: A systematic sequencing-based approach for microbial contaminant detection and functional inference
Source: BMC Biol. 2019 Sep 13;17:72. doi: 10.1186/s12915-019-0690-0 (PMC6743104; doi:10.1186/s12915-019-0690-0)
Supplement: Supplementary file 1 — Figure S1. Results of the reversion test employing different parameters for Bowtie2. Using the simulated read sets created in Fig. 1b, Bowtie2 was performed with the parameters “--very-sensitive” (A), “--fast” (B), and “--very-fast” (C). (D) Distribution of the reverted reads of 5709 species at genus-level resolution (“--sensitive” parameter). Figure S2. Results of the reversion test in the three existing pipelines. (A) FDR distributions at genus-level resolution. (B) Distribution of the reverted reads of 5709 species at genus-level resolution. Additional file 2 details how these values were calculated. Figure S3. Examples of the scoring function used to weight multi-genera-hit reads. The slope of the exponential function is defined by the overall mapping status of the input reads incorporated into M (the total number of microbe-mapped reads) and N (the total number of unique or multiple hits of all microbe-mapped reads). For instance, a read of ENCSR000AAR that mapped to ten distinct genera (T = 10) is counted as 0.4. Figure S4. Profiling contamination prevalence in public RNA-seq datasets. (A) Distributions of the fractions of microbe-mapped reads in the total input reads of ENCODE and IHBM2 (Illumina Human BodyMap 2.0). (B) Frequencies of 240 microbial genera detected as significant contaminants in the samples. The gray-colored bars represent known contaminants reported in Salter, et al., 2014 [12]. Microbes labeled in black-, blue-, and red correspond to bacterium, fungus, and virus, respectively. Figure S5. Results of the enrichment analysis of GO biological process terms with DEGs found in Myco(−) hBM-MSC BM1 and BM2. In BM1 and BM2, 2237 DUGs (differentially upregulated genes) and 1301 DUGs were identified, respectively. The heatmap showed over-enriched GO terms in both BM1 and BM2. The enrichment analysis of the reactome showed no significant enrichments (q-value < 0.001). DUG_BM1: differentially-upregulated genes in Myco(−) hBM-MSCs that were sequenced in t [file 12915_2019_690_MOESM1_ESM.pdf]

Figure S1

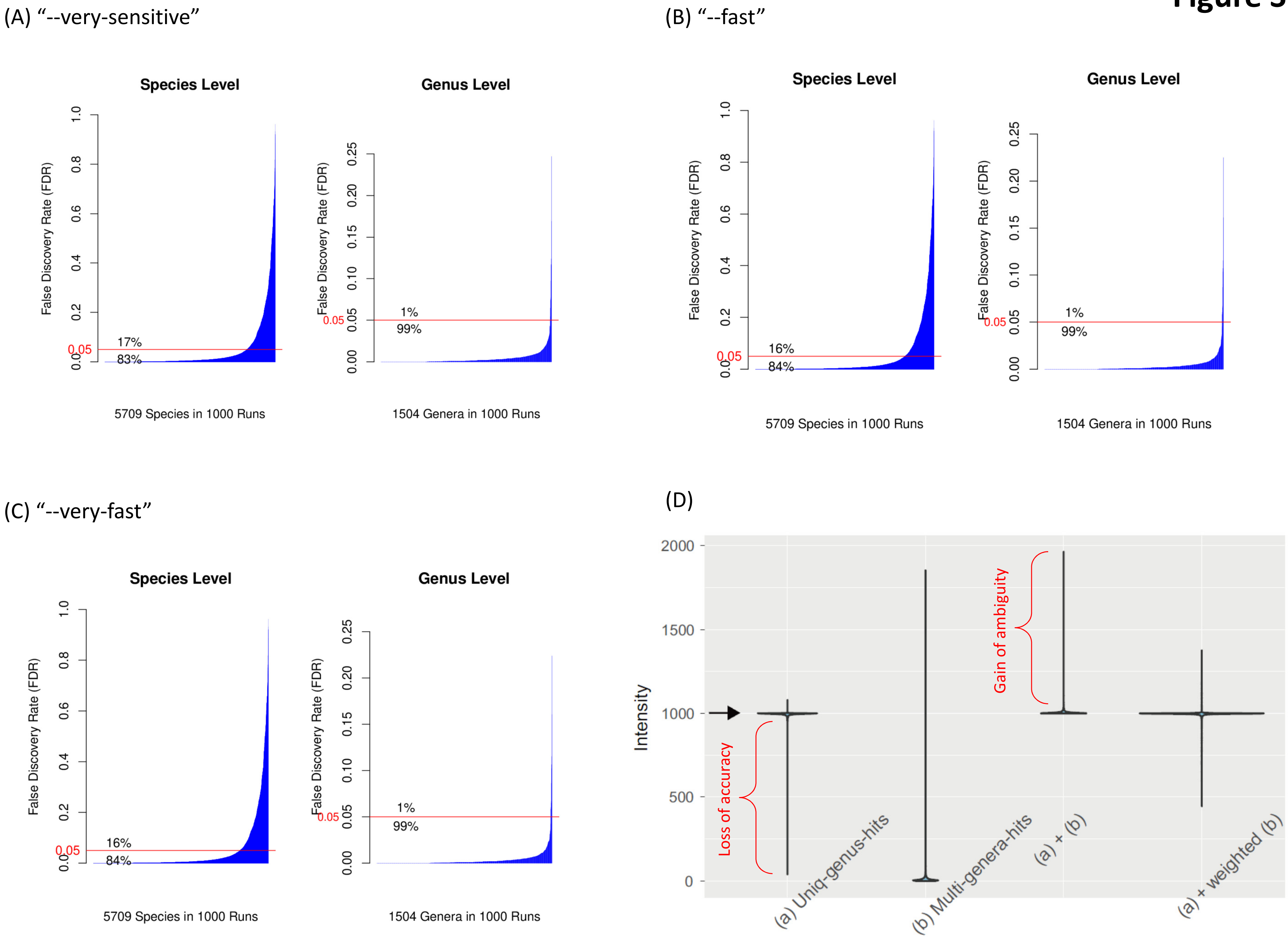
(A) “--very-sensitive”
Species Level
Genus Level
False Discovery Rate (FDR)
17%
83%
0.05
1%
99%
5709 Species in 1000 Runs
1504 Genera in 1000 Runs
(B) “--fast”
Species Level
Genus Level
False Discovery Rate (FDR)
16%
84%
0.05
1%
99%
5709 Species in 1000 Runs
1504 Genera in 1000 Runs
(C) “--very-fast”
Species Level
Genus Level
False Discovery Rate (FDR)
16%
84%
0.05
1%
99%
5709 Species in 1000 Runs
1504 Genera in 1000 Runs
(D)
Intensity
Gain of ambiguity
Loss of accuracy
(a) Uniq-genus-hits
(b) Multi-genera-hits
(a) + (b)
(a) + weighted (b)

Figure S2

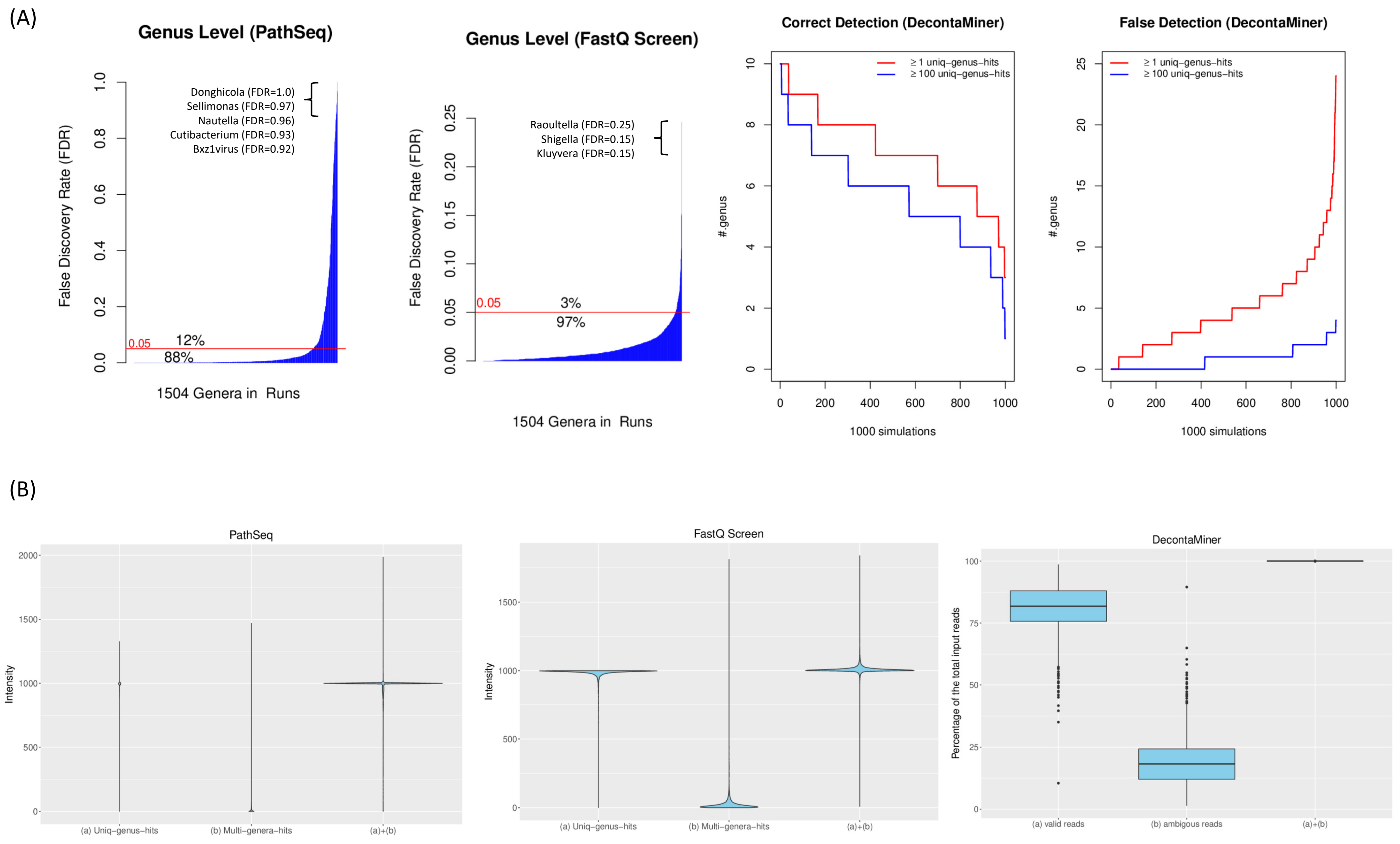

(A)
Genus Level (PathSeq)
False Discovery Rate (FDR)
0.0
0.2
0.4
0.6
0.8
1.0
Donghicola (FDR=1.0)
Sellimonas (FDR=0.97)
Nautella (FDR=0.96)
Cutibacterium (FDR=0.93)
Bxz1virus (FDR=0.92)
0.05
12%
88%
1504 Genera in Runs
Genus Level (FastQ Screen)
0.00
0.05
0.10
0.15
0.20
0.25
Raoultella (FDR=0.25)
Shigella (FDR=0.15)
Kluyvera (FDR=0.15)
3%
97%
Correct Detection (DecontaMiner)
False Detection (DecontaMiner)
#.genus
≥ 1 uniq−genus−hits
≥ 100 uniq−genus−hits
1000 simulations
(B)
PathSeq
FastQ Screen
DecontaMiner
Intensity
Percentage of the total input reads
(a) Uniq−genus−hits
(b) Multi−genera−hits
(a)+(b)
(a) valid reads
(b) ambigous reads

Figure S3

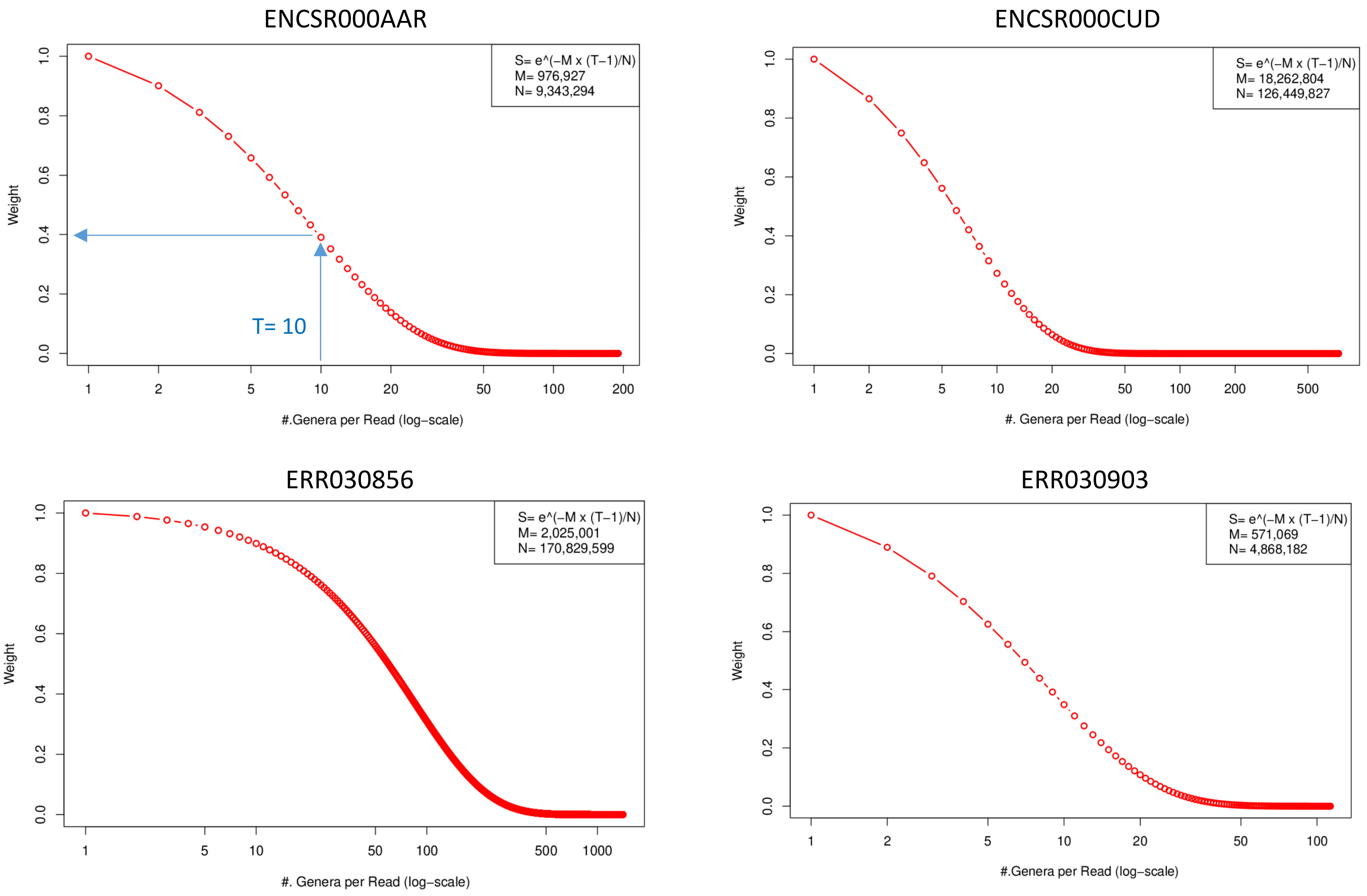
ENCSR000AAR
S= e^(−M x (T−1)/N)
M= 976,927
N= 9,343,294
T= 10
Weight
#.Genera per Read (log−scale)
ENCSR000CUD
S= e^(−M x (T−1)/N)
M= 18,262,804
N= 126,449,827
Weight
#. Genera per Read (log−scale)
ERR030856
S= e^(−M x (T−1)/N)
M= 2,025,001
N= 170,829,599
Weight
#. Genera per Read (log−scale)
ERR030903
S= e^(−M x (T−1)/N)
M= 571,069
N= 4,868,182
Weight
#.Genera per Read (log−scale)

Figure S4

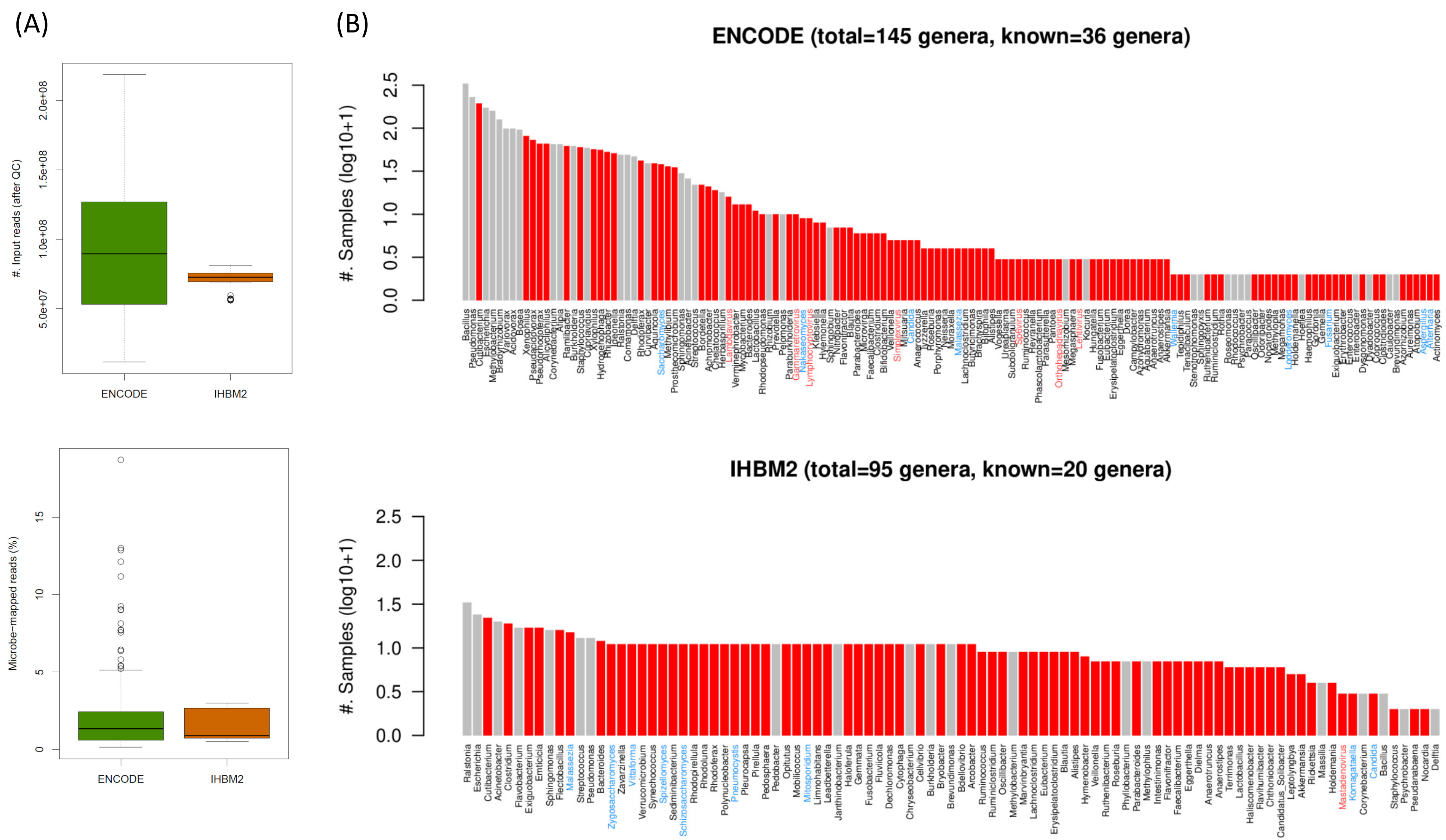
(A)
(B)
#. Input reads (after QC)
5.0e+07
1.0e+08
1.5e+08
2.0e+08
ENCODE
IHBM2
Microbe−mapped reads (%)
0
5
10
15
ENCODE
IHBM2
ENCODE (total=145 genera, known=36 genera)
IHBM2 (total=95 genera, known=20 genera)
#. Samples (log10+1)
0.0
0.5
1.0
1.5
2.0
2.5

Figure S5

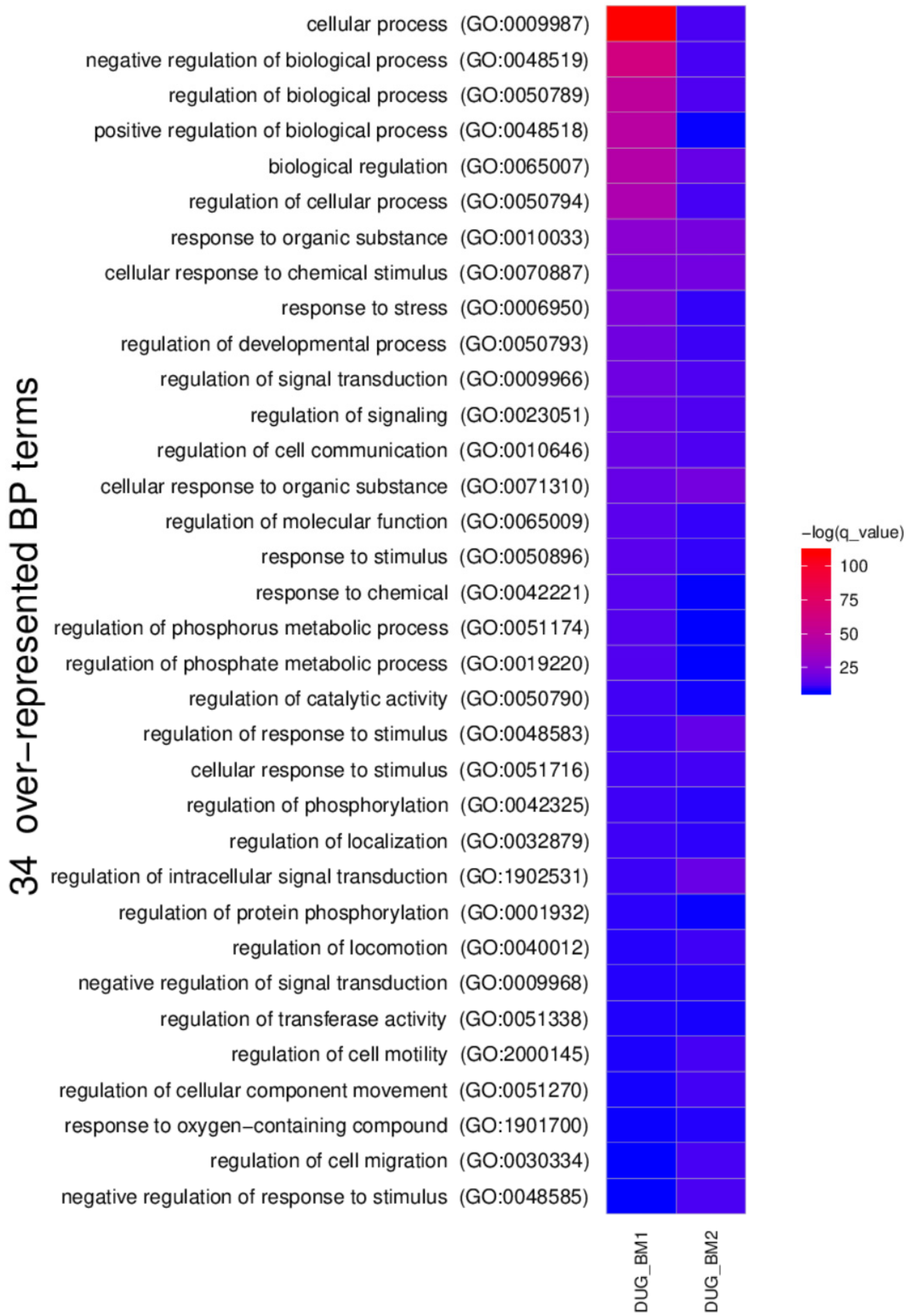

34 over–represented BP terms
cellular process (GO:0009987)
negative regulation of biological process (GO:0048519)
regulation of biological process (GO:0050789)
positive regulation of biological process (GO:0048518)
biological regulation (GO:0065007)
regulation of cellular process (GO:0050794)
response to organic substance (GO:0010033)
cellular response to chemical stimulus (GO:0070887)
response to stress (GO:0006950)
regulation of developmental process (GO:0050793)
regulation of signal transduction (GO:0009966)
regulation of signaling (GO:0023051)
regulation of cell communication (GO:0010646)
cellular response to organic substance (GO:0071310)
regulation of molecular function (GO:0065009)
response to stimulus (GO:0050896)
response to chemical (GO:0042221)
regulation of phosphorus metabolic process (GO:0051174)
regulation of phosphate metabolic process (GO:0019220)
regulation of catalytic activity (GO:0050790)
regulation of response to stimulus (GO:0048583)
cellular response to stimulus (GO:0051716)
regulation of phosphorylation (GO:0042325)
regulation of localization (GO:0032879)
regulation of intracellular signal transduction (GO:1902531)
regulation of protein phosphorylation (GO:0001932)
regulation of locomotion (GO:0040012)
negative regulation of signal transduction (GO:0009968)
regulation of transferase activity (GO:0051338)
regulation of cell motility (GO:2000145)
regulation of cellular component movement (GO:0051270)
response to oxygen–containing compound (GO:1901700)
regulation of cell migration (GO:0030334)
negative regulation of response to stimulus (GO:0048585)
DUG_BM1
DUG_BM2
−log(q_value)
100
75
50
25

Figure S6

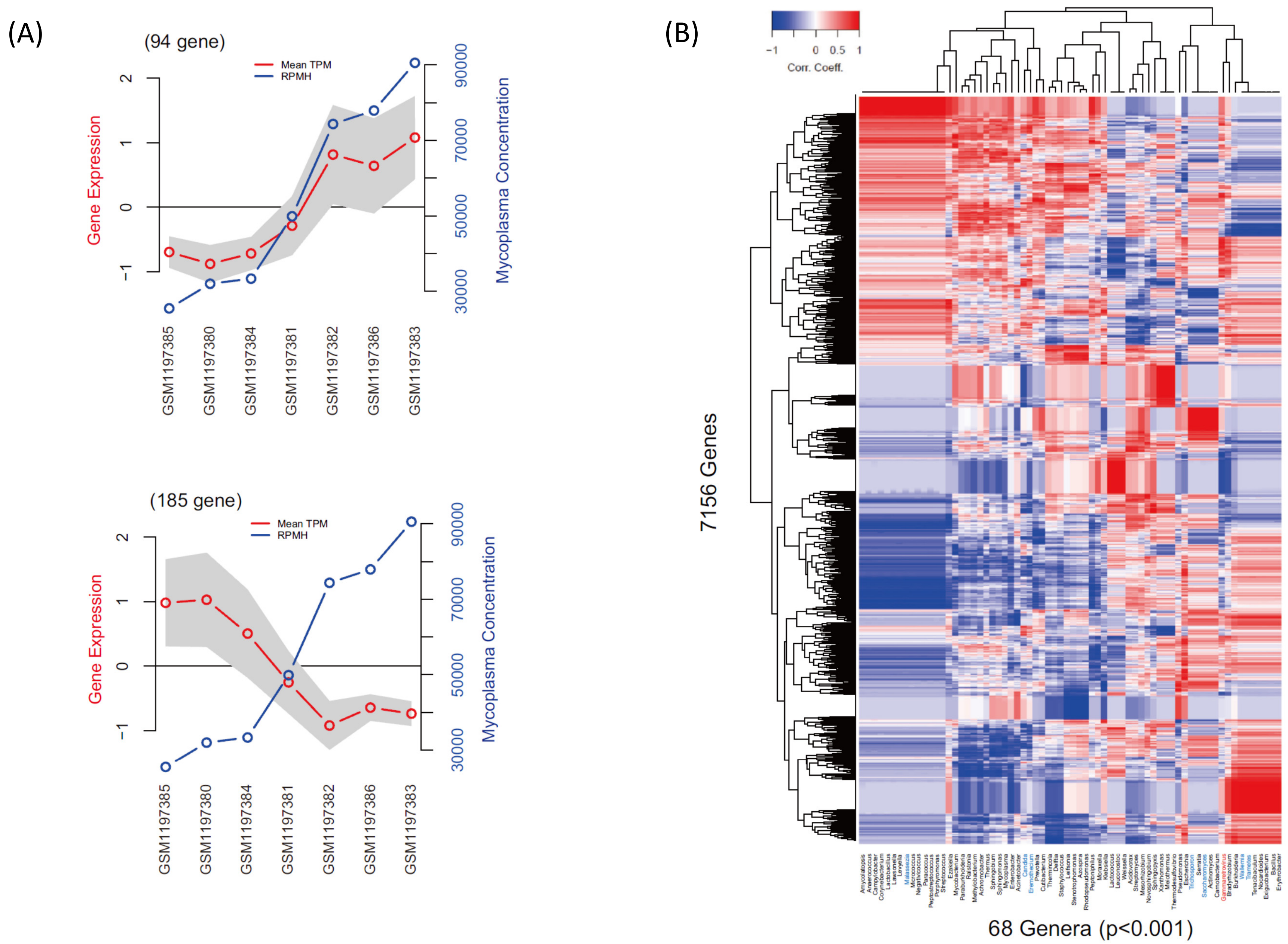

(A)
(94 gene)
Mean TPM
RPMH
Gene Expression
Mycoplasma Concentration
2
1
0
−1
30000
50000
70000
90000
GSM1197385
GSM1197380
GSM1197384
GSM1197381
GSM1197382
GSM1197386
GSM1197383
(185 gene)
Mean TPM
RPMH
Gene Expression
Mycoplasma Concentration
GSM1197385
GSM1197380
GSM1197384
GSM1197381
GSM1197382
GSM1197386
GSM1197383
(B)
−1
0
0.5
1
Corr. Coeff.
7156 Genes
68 Genera (p<0.001)

Figure S7

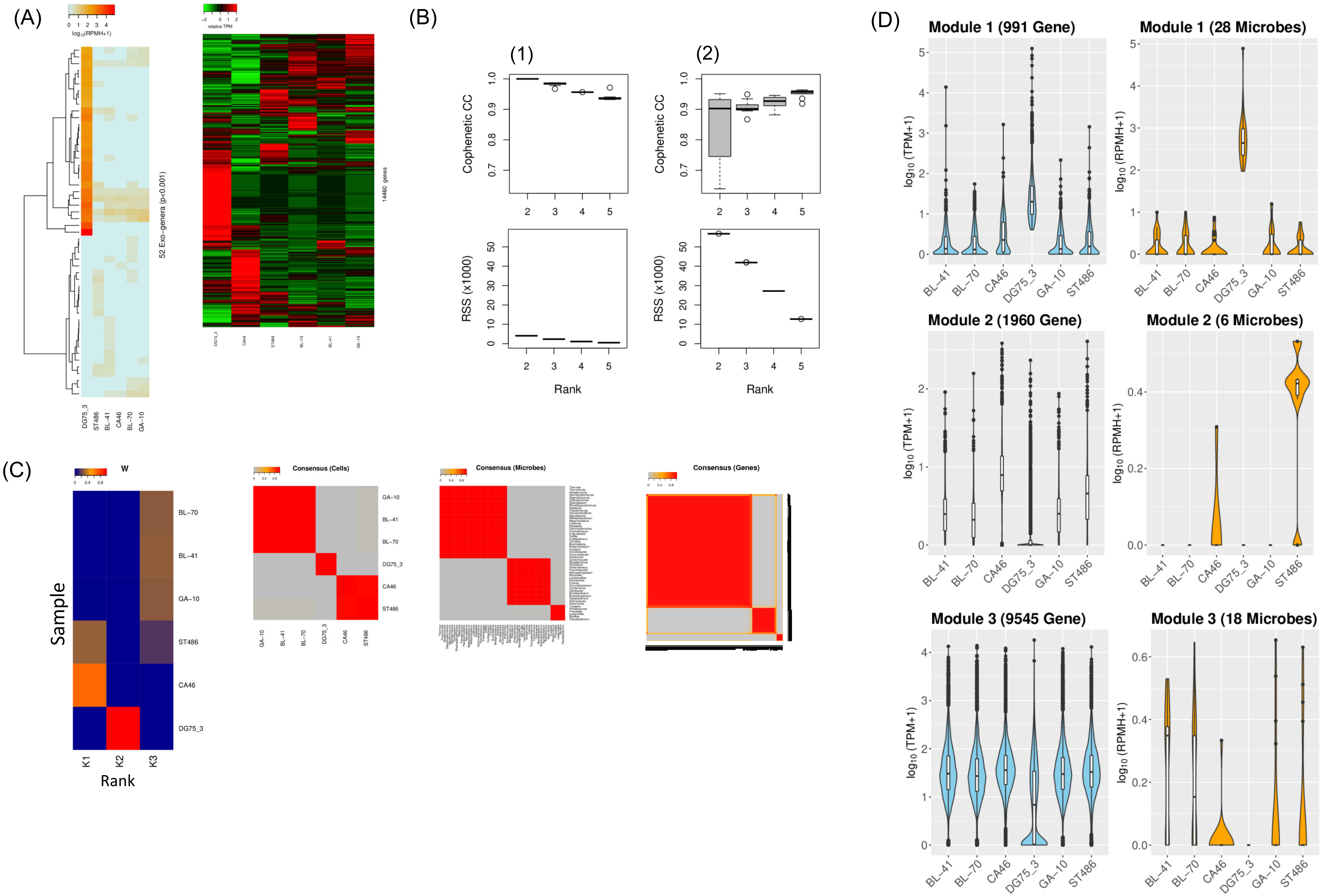
(A)
log10(RPMH+1)
relative TPM
52 Exo-genera (p<0.001)
14460 genes
DG75_3
ST486
BL-41
CA46
BL-70
GA-10
(B)
(1)
(2)
Cophenetic CC
RSS (x1000)
Rank
(C)
W
Sample
Rank
K1
K2
K3
BL-70
BL-41
GA-10
ST486
CA46
DG75_3
Consensus (Cells)
Consensus (Microbes)
Consensus (Genes)
(D)
Module 1 (991 Gene)
Module 1 (28 Microbes)
Module 2 (1960 Gene)
Module 2 (6 Microbes)
Module 3 (9545 Gene)
Module 3 (18 Microbes)
log10 (TPM+1)
log10 (RPMH+1)
